# USH2A Gene Mutations in Rabbits Lead to Progressive Retinal Degeneration and Hearing Loss

**DOI:** 10.1167/tvst.12.2.26

**Published:** 2023-02-16

**Authors:** Van Phuc Nguyen, Jun Song, Diane Prieskorn, Junhuang Zou, Yanxiu Li, David Dolan, Jie Xu, Jifeng Zhang, K. Thiran Jayasundera, Jun Yang, Yehoash Raphael, Naheed Khan, Michael Iannuzzi, Charles Bisgaier, Y. Eugene Chen, Yannis M. Paulus, Dongshan Yang

**Affiliations:** 1 Center for Advanced Models for Translational Sciences and Therapeutics, University of Michigan, Ann Arbor, MI, USA; 2 W.K. Kellogg Eye Center, Department of Ophthalmology and Visual Sciences, University of Michigan, Ann Arbor, MI, USA; 3 Kresge Hearing Research Institute, Department of Otolaryngology-Head and Neck Surgery, University of Michigan, Ann Arbor, MI, USA; 4 John A. Moran Eye Center, Department of Ophthalmology, University of Utah Medical School, Salt Lake City, UT, USA; 5 GeneToBe Inc, Ann Arbor, MI, USA

**Keywords:** UHS2A mutation gene, CRISPR/cas9, usher syndrome, electroretinography, photoreceptor degeneration

## Abstract

**Purpose:**

Mutations in USH2A gene are responsible for the greatest proportion of the Usher Syndrome (USH) population, among which more than 30% are frameshift mutations on exon 13. A clinically relevant animal model has been absent for USH2A-related vision loss. Here we sought to establish a rabbit model carrying USH2A frameshift mutation on exon 12 (human exon 13 equivalent).

**Methods:**

CRISPR/Cas9 reagents targeting the rabbit USH2A exon 12 were delivered into rabbit embryos to produce an USH2A mutant rabbit line. The USH2A knockout animals were subjected to a series of functional and morphological analyses, including acoustic auditory brainstem responses, electroretinography, optical coherence tomography, fundus photography, fundus autofluorescence, histology, and immunohistochemistry.

**Results:**

The USH2A mutant rabbits exhibit hyper-autofluorescent signals on fundus autofluorescence and hyper-reflective signals on optical coherence tomography images as early as 4 months of age, which indicate retinal pigment epithelium damage. Auditory brainstem response measurement in these rabbits showed moderate to severe hearing loss. Electroretinography signals of both rod and cone function were decreased in the USH2A mutant rabbits starting from 7 months of age and further decreased at 15 to 22 months of age, indicating progressive photoreceptor degeneration, which is confirmed by histopathological examination.

**Conclusions:**

Disruption of USH2A gene in rabbits is sufficient to induce hearing loss and progressive photoreceptor degeneration, mimicking the USH2A clinical disease.

**Translational Relevance:**

To our knowledge, this study presents the first mammalian model of USH2 showing the phenotype of retinitis pigmentosa. This study supports the use of rabbits as a clinically relevant large animal model to understand the pathogenesis and to develop novel therapeutics for Usher syndrome.

## Introduction

Usher syndrome (USH) is an autosomal-recessive genetic disorder resulting in hearing loss, progressive visual impairment, and, in some types, balance issues.^1^ The major ocular symptom of patients with USH is a disease called retinitis pigmentosa (RP). RP causes the light-sensing photoreceptor cells in the retina to gradually deteriorate, initially resulting in night blindness, followed by tunnel vision, and severe, permanent, progressive vision loss. More than 400,000 people are affected by USH worldwide, accounting for approximately 50% of all hereditary deaf-blindness cases.^2^ USH is classified into three subtypes (I, II, and III), which are distinguished by severity and age at onset of deafness, presence or absence of vestibular dysfunction, and age at onset of RP. Among them, type II (USH2) is the most common subtype, characterized by hearing loss from birth and progressive vision loss that begins in adolescence or adulthood. USH2 may be caused by mutations in any of three genes: USH2A, GPR98, and DFNB31, with USH2A mutations being the most prevalent, present in approximately 70% of USH2 cases.^3^ Mutations in the USH2A gene are also a cause of some forms of RP without hearing loss (i.e., nonsyndromic RP).^4^ More than 700 pathogenic USH2A mutations have been identified, as reported in the LOVD database (http://www.lovd.nl). Mutations in exon 13 account for more than 30% of all USH2A cases, including the two most recurrent mutations in USH2A, c.2299delG (p. Glu767fs*21) and c.2276G>T (p. Cys759Phe).^5^ Despite extensive research, there is currently no cure for USH2. Hearing aids provide benefits to USH2 patients who have moderate to severe hearing loss; however, efforts to mitigate the progressive vision loss caused by RP have been less effective. Most individuals with USH2 RP will eventually suffer from blindness. Therefore, there is a pressing unmet clinical need to develop novel therapeutics for USH2.

Mouse and zebrafish models of USH2A have been developed. Unfortunately, phenotypes observed in the retinae of USH2A patients are not faithfully replicated in mouse models carrying USH2A mutations.^6^^–^^8^ USH2A knockout (KO) mice suffer from hearing loss, but only manifest weak and very late-onset retinal degeneration phenotype. The zebrafish models exhibit early retinal degeneration phenotypes. However, their features of adult photoreceptor regeneration as well as the phylogenetic separation from humans may pose problems in translational studies.^9^ Therefore, development of an alternative mammalian model of USH2, which more closely approximates human physiology, function, and anatomy, and importantly RP pathogenesis is of prime importance and may accelerate translating discoveries from animal models into clinical therapies and interventions for the disease.

Rabbits, compared with mice, are closer to humans in terms of phylogenesis, anatomic features, physiology, and pathophysiological responses^10^^–^^14^ and are used as a classic laboratory animal species to develop novel therapeutics for humans and refine medical and surgical equipment.^13^^,^^15^^–^^19^ Historically, retinal degeneration has been studied in rhodopsin Pro347Leu transgenic rabbits, a model of RP.^20^^–^^31^ Recently, the emerging gene editing technology in rabbits has greatly increased their value to biomedicine, motivating our efforts to develop rabbits that carrying the disease causing mutations found in human patients, as models to replicate human diseases more precisely.^32^^–^^34^ In this study, we report the development of USH2A rabbits by CRISPR/Cas9. This novel model is expected to greatly facilitate both the basic and translational studies of USH.

## Materials and Methods

### Animals

New Zealand White (NZW) rabbits were purchased from Covance (Princeton, NJ) or Charles River Labs (Bar Harbor, ME). The animal maintenance, care and use procedures were reviewed and approved by the Institutional Animal Care and Use Committee of the University of Michigan. All the animals were housed in a specific pathogen free and air-conditioned room with a 12-hour light–dark cycle. The light fluence in the animal room was maintained at a mean of 61.08 Lux (top cages), 33.93 Lux (mid cages), and 21.11 Lux (floor cages). The animal was allowed free access to the water and fed with standard laboratory food. All procedures were carried out in accordance with the approved guidelines and were performed in accordance with the ARVO Statement for the Use of Animals in Ophthalmic and Vision Research. Before each experiment procedure, rabbits were anesthetized by intramuscular injection of a mixed solution of ketamine (40 mg/kg, 100 mg/mL) plus xylazine (5 mg/kg, 100 mg/mL). All the animal pupils were dilated by receiving a drop of 1% tropicamide and 2.5% phenylephrine hydrochloride ophthalmic. One drop of 0.5% topical tetracaine was added to each eye before treatment for topical anesthesia. All efforts were made to minimize any discomfort.

### Reverse Transcription Polymerase Chain Reaction (PCR) and Real-time PCR Analysis

Total RNA from retina, sclera, brain, liver, kidney, and bone marrow were isolated using the RNeasy kit (Qiagen, Hilden, Germany). Reverse transcription was used to generate cDNA (SuperScript III First-Strand Synthesis System, Thermo Fisher Scientific [Waltham, MA], 18080-05) as template for reverse transcription PCR and real time PCR. For real-time PCR analysis, samples were analyzed on a BioRad CFX Connect Real-Time PCR Detection System and amplification was detected using the SYBR green method (BioRad, Hercules, CA; SYBR green supermix). PCR primers (RTF1/RTR1, RTF2/RTR2) are listed in Table S2. Rabbit 18S rRNA or GAPDH expression was used as internal control. The reverse transcription PCR products were purified and subject to Sanger sequencing.

### Histopathology and Immunofluorescent Staining

To euthanize the rabbits, euthanasia solution (Euthanasia, 0.22 mg/kg, 50 mg/mL, VetOne, Boise, ID) was injected into the rabbit intravenously through the marginal ear vein. The eyeballs were harvested and fixed in Davidson's fixative solution for 24 hours. The eye cup was then embedded in paraffin. The sample was sectioned to a thickness of 4 µm using a Leica Autostainer XL (Leica Biosystems, Nussloch, Germany) and stained with hematoxylin and eosin (H&E). The H&E slides were observed using a Leica DM600 light microscope (Leica Biosystems) and the images were captured using a BF450C camera.

For immunostaining, 5-mm punches were taken from fresh rabbit retinae at the central vertical line 2 mm ventral to the optic nerve root, frozen immediately in the Tissue-Tek optimal cutting temperature (OCT) compound (Sakura Finetek USA, Inc, Torrance, CA) and sectioned at 12 µm using Leica CM3050S cryostat. The retinal sections were fixed by 4% formaldehyde in phosphate-buffered saline (PBS) for 10 minutes, permeabilized by 0.1% Triton X-100/PBS for 5 minutes and blocked in 5% goat serum and PBS for 1 hour. The retinal sections were then incubated with usherin antibody^35^ at 4 °C overnight. After several washes with PBS, the retinal sections were incubated with a mixture of Alexa Fluor 488–conjugated peanut agglutinin, Alexa Fluor 568–conjugated goat anti-rabbit secondary antibody, and Hoechst dye 33342 (Thermo Fisher Scientific) for 1 hour. The immunofluorescence images were captured using a Leica SP8 confocal microscope with a HC PL APO 63 × 1.40 OIL CS2 objective.

### CRISPR Reagents

The Cas9 expression plasmid JDS246 was obtained from Addgene. Cas9 messenger RNA (mRNA) was transcribed in vitro, capped and polyadenylated using the T7 mScript Standard mRNA Production System (C-MSC100625; CELLSCRIPT, Madison, WI). Guide RNA (gRNA) was designed using CRISPOR software,^36^ synthesized as chemically modified (2ʹ-O-methyl at 3 first and last bases, 3ʹ phosphorothioate bonds between first three and last two bases) single-strand gRNA (sgRNA) (EZ Kit, Synthego, Redwood City, CA). The target sequence on rbUSH2A is shown in Fig. 2A and Table S1. Cas9 mRNA and sgRNA were diluted in RNase-free TE buffer (1 mM Tris-Cl, pH 8.0, 0.1 mM EDTA), stored at –80 °C in 10 µL aliquots, and were thawed and kept on ice before microinjection.

### Rabbit Genome Editing

Methods of rabbit genome editing have been described previously in detail.^37^ Briefly, pronuclear stage rabbit embryos were injected with approximately 2 to 5 pl RNase-free TE buffer (1 mM Tris-Cl, pH 8.0, 0.1 mM EDTA) containing 150 ng/µL Cas9 mRNA and 50 ng/µL sgRNA. Injected embryos were washed three times in embryo culture medium, which consisted of Earle's Balanced Salt Solution (E2888, Sigma, St Louis, MO) supplemented with nonessential amino acids (M7145, Sigma), essential amino acids (B-6766, Sigma), 1 mM l-glutamine (25030-081, Life Technologies, Grand Island, NY), 0.4 mM sodium pyruvate (11360–070, Life Technologies), and 10% fetal bovine serum. Twenty to thirty embryos were transferred surgically to oviducts of each synchronized recipient doe. For gRNA validation, instead of transferring to recipients, the injected embryos were washed and cultured in the medium at 38.5 °C in 5% CO_2_ for additional 3 days until they reach blastocyst stage.

### Detection of Gene Editing Events

For gRNA in vivo validation, injected embryos developed to blastocyst stage in culture were collected in 1.5 µL water individually and the whole genome was replicated using a REPLI-g Mini Kit (Qiagen, Germantown, MD) following the manufacturer's protocol. For rabbit genotyping, genomic DNA was isolated from the newborn kits ear skin. Genomic DNA was then amplified by PCR using corresponding primers (Table S2): primers for on target mutation detection, F1/R1 and F2/R2; primers for next-generation sequencing, dsF/dsR; primers for off-target 1 (OT1) detection, OT1F/OT1R; primers for off-target 2 (OT2) detection, OT2F/OT2R; primers for off-target 3 (OT3) detection, OT3F/OT3R. PCR products were purified and subjected to T7E1 assay, Sanger sequencing, and next-generation sequencing (MGH CCIB DNA core). Next-generation sequencing data were analyzed using CRISPResso2 software.^38^

### Rabbit Eye Examination and Imaging Procedure

A comprehensive examination of the eyelids, conjunctiva, cornea, anterior chamber, iris, and lens was performed before imaging by slit lamp bio-microscopy (SL120, Carl Zeiss, Jena, Germany). Fundus photography, fundus autofluorescence, fluorescein angiography, and indocyanine green angiography were used to evaluate the vascular network of the retina and choroid. Briefly, after pupil dilation, a clinical fundus camera (TRC-50EX, Topcon Corporation, Tokyo, Japan) was used to acquire fundus photography, fundus autofluorescence, fluorescein angiography, and indocyanine green angiography. Fluorescein sodium (0.2 mL, 10% solution) (Akorn, Lake Forest, IL) and indocyanine green (0.5 mg/kg, 5 mg/mL, HUB Pharmaceuticals LLC, Patheon, Italy) were injected in the rabbit marginal ear vein. Photographs were captured immediately after injection up to 10 minutes to capture early, middle, and late phase angiography images. There was a 5-minute interval between the two kinds of angiography tests.

Spectral domain OCT imaging was performed using a Ganymede-II-HR OCT system (Thorlabs, Newton, NJ) with modification as described previously.^39^ Briefly, two super luminescent light-emitting diodes with a center wavelength of 905 nm were used to illuminate the surface of the cornea and focused on the fundus by the rabbit eye optics. The average power of the OCT probing light was 0.8 mW. The aerial lateral and axial resolutions are 3.8 µm and 4.0 µm, respectively. The system can achieve an imaging depth of 1.9 mm. The rabbits were put on a custom-built platform, and the eye position was adjusted under the ophthalmic lens using a CCD camera to visualize the region of interest.

### Electroretinography (ERG)

Full-field ERG was performed after pupillary dilation. After 60 minutes of dark adaptation, rabbits were anesthetized. After topical anesthesia, ERG-Jet contact lens electrodes (The Electrode Store, Enumclaw, WA) were applied. Corneal hydration was maintained with a 2.5% hypromellose ophthalmic demulcent solution (Akorn Inc). A pair of reference electrodes and a ground electrode (needle electrodes, The Electrode Store) were placed subcutaneously behind the bilateral ears and in the scruff, respectively. All animal handling was done under dim red light. ERGs were recorded with a Ganzfeld configuration using the LKC UTAS 3000 electrophysiology system (LKC Technologies, Gaithersburg, MD). ERG responses were amplified at 2500 gain at 0.312 to 500.000 Hz and digitized at a rate of 2000 Hz. Scotopic ERGs were recorded at a dim flash intensity of 0.01 cd.s/m^2^ to obtain the rod isolated ERG and at 3.0 cd.s/m^2^ to obtain the combined rod–cone ERG. After 10 minutes of light adaptation to a white 32 cd/m^2^ rod-suppressing background, photopic ERGs were recorded at a flash intensity of 3.0 cd. s/m^2^. For ERG analyses, the a-wave amplitude was measured from the prestimulus baseline to the trough of the a-wave, and the implicit time of the a-wave was measured from flash onset to the trough of the a-wave. The b-wave amplitude was measured from the trough of the a-wave to the peak of the b-wave, and the b-wave implicit time was measured from flash onset to the peak of the b-wave. ERG recording was performed using a xenon white flash, 1000 Hz sampling frequency, 0.312 to 300.000 Hz cut off filter, 500-ms recording time, 10-ms baseline before the flash, and no notch filter was used.

### Acoustic Auditory Brainstem Responses (ABR)

The auditory sensitivity of the animals was evaluated by recording ABRs to acoustic stimuli. Rabbits were anesthetized intramuscularly with xylazine (3–5 mg/kg) and ketamine (40 mg/kg) and placed on a warm water-circulating heating in a sound attenuated chamber. Supplemental oxygen via a nose cone and warm subcutaneous saline (5–10 mg/kg) was provided. The heart rate, oxygen saturation, and temperature were monitored throughout the testing period. Needle electrodes were placed subcutaneously under each pinna: test ear (reference), contralateral ear (ground), and at the vertex (active) of the animal's head to record the neural output. Tucker Davis Technologies System III hardware and SigGen/BioSig software (Tucker Davis Technologies, Alachua, FL) were used to present the stimulus and record responses. Tones were delivered through a single channel of a modified in-ear headphone (JBL Endurance Run, 8.2-mm dynamic driver) placed in the ear canal with the ear tip on. Stimulus presentation was 15-ms tone bursts, with 1-ms rise/fall times, presented 10 per second. Up to 1024 responses were averaged for each stimulus level. Responses were collected for stimulus levels in 10-dB steps at higher stimulus levels, with additional 5-dB steps near threshold. Thresholds were interpolated between the lowest stimulus level where a response was observed, and 5 dB lower, where no response is observed. ABR thresholds and suprathresholds were tested at three frequencies (4, 12, and 16 kHz). After the procedure, the xylazine reversal agent, atipamezole (1 mg/kg), was administered subcutaneously to speed recovery and rapidly improve oxygen saturation levels, without the need of supplemental oxygen. Rabbits were kept warm until ambulatory.

## Results and Discussion

### Usherin Is Highly Conserved in Rabbit and Human

**Figure 1.**
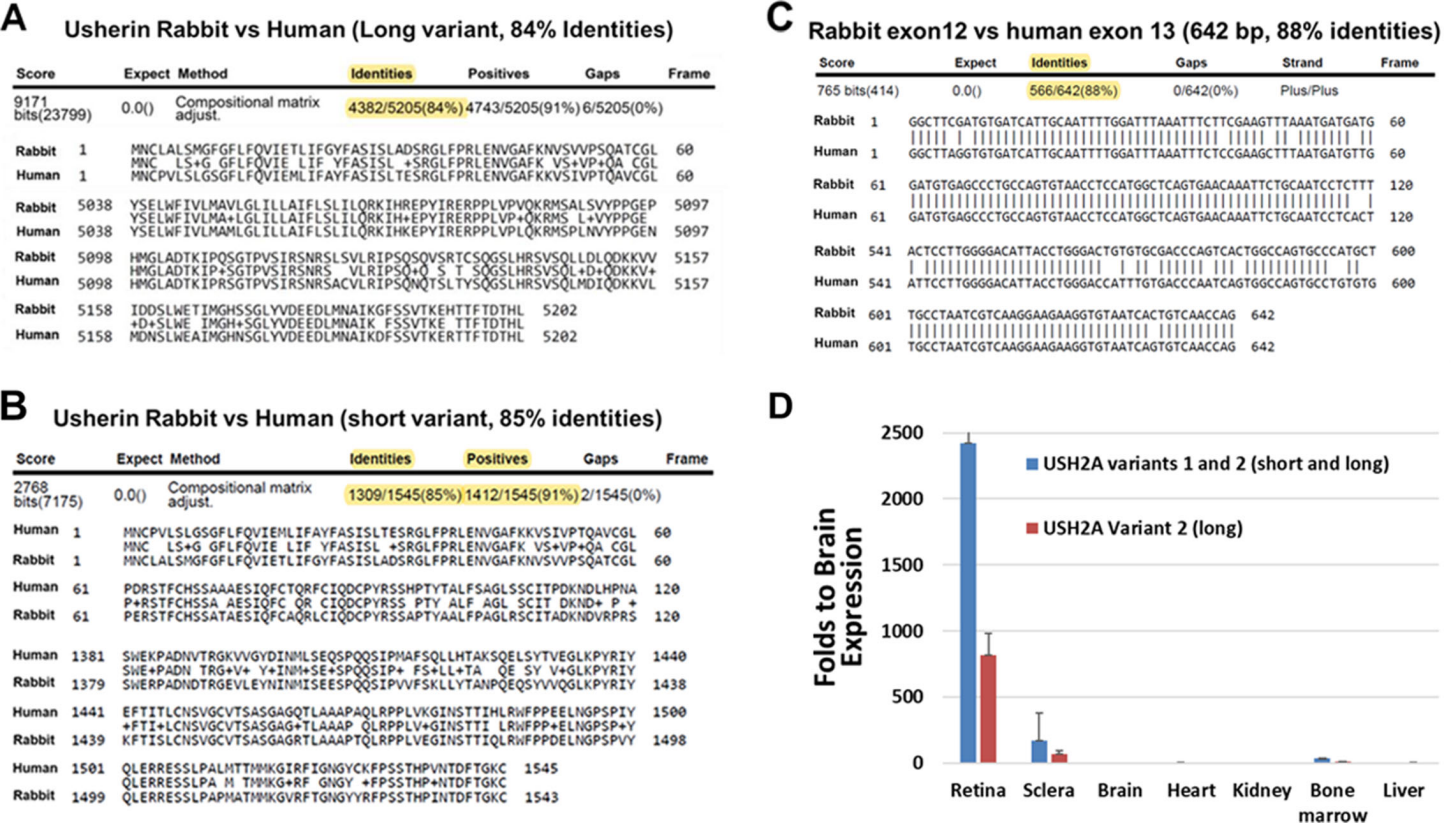
USH2A is conserved in rabbit and human. **(A)** Rabbit versus human Usherin protein long isoform sequence alignment using BlastP program. **(B)** Rabbit versus human Usherin protein short isoform sequence alignment using BlastP program. **(C)** cDNA sequence alignment of rabbit USH2A exon12 versus human USH2A exon13. **(D)** Real-time PCR detection of USH2A variants in adult rabbit organs. Primer pair 1 (RTF1, RTR1) detects both variant 1 and variant 2 (v1+v2, blue bar); Primer pair 2 (RTF2, RTR2) detects variant 2 only (*purple bar*). Values were normalized to18S rRNA expression. The *y* axes show the fold change relative to the expression level in brain. Error bars represent standard deviation (*n* = 3).

The rabbit USH2A gene has two isoforms: (i) the short transcript containing 23 exons that encodes 1543 amino acids (ensemble transcript ID: ENSOCUT00000014751.4); and (ii) the long transcript contains 74 exons encodes 5202 amino acids (NCBI reference sequence: XM_008268426.2). Analysis of protein sequences of usherin revealed that Usherin is highly conserved in human and rabbit, with 84% identify score for long isoforms and 85% identity score for short isoforms, and 91% positive score for both isoforms (Figs. 1A, B). As shown in Fig. 1C, rabbit exon12 of USH2A is the same as the length of human exon13—both are 642 bp long—which notably is an in-frame length that is suitable for testing the exon deletion-based therapy.^40^ We examined the expression profiles of USH2A gene in rabbits by real-time PCR using two pairs of primers: (i) pair 1 on exon11 and exon12, that detects both short and long isoforms, and (ii) pair 2 on exon50 and exon51, that detects only the long isoform. Fig. 1D shows both isoforms of USH2A are highly expressed in the rabbit retina, but barely detected in other organs or tissues examined.

### Production of USH2A-Mutant Rabbits

**Figure 2.**
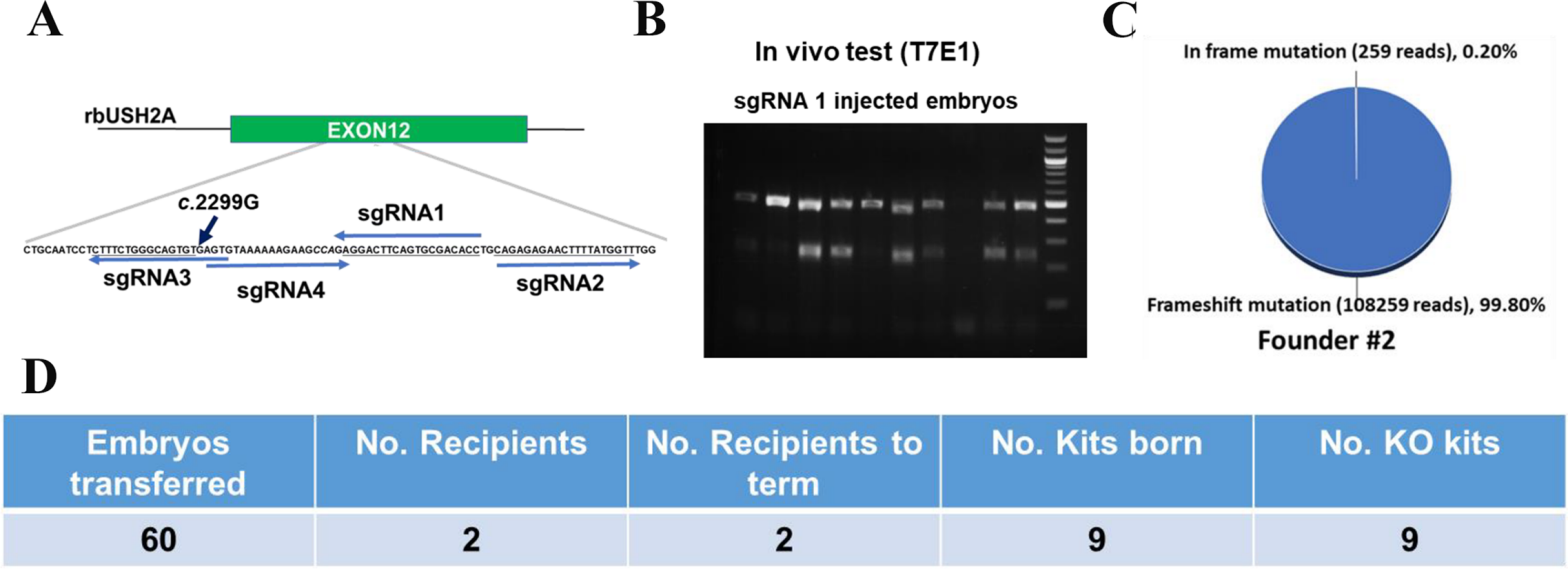
Production of USH2A mutant rabbits. **(A)** Illustration of the CRISPR/Cas9–mediated targeting strategy to produce rbUSH2A mutant rabbits. **(B)** sgRNA1 was validated in rabbit embryos using T7E1 analysis. Each lane representative one injected embryos. The PCR product of 479 bp will be cleaved into 238 bp and 241 bp bands if the embryos have indels generated at the gRNA target. M, NEB 100 bp DNA ladder. **(C)** Representative next-generation sequencing analysis of the founder rabbits ear skin biopsy showed high frequencies of frameshift mutations **(D)**.

**Figure 3.**
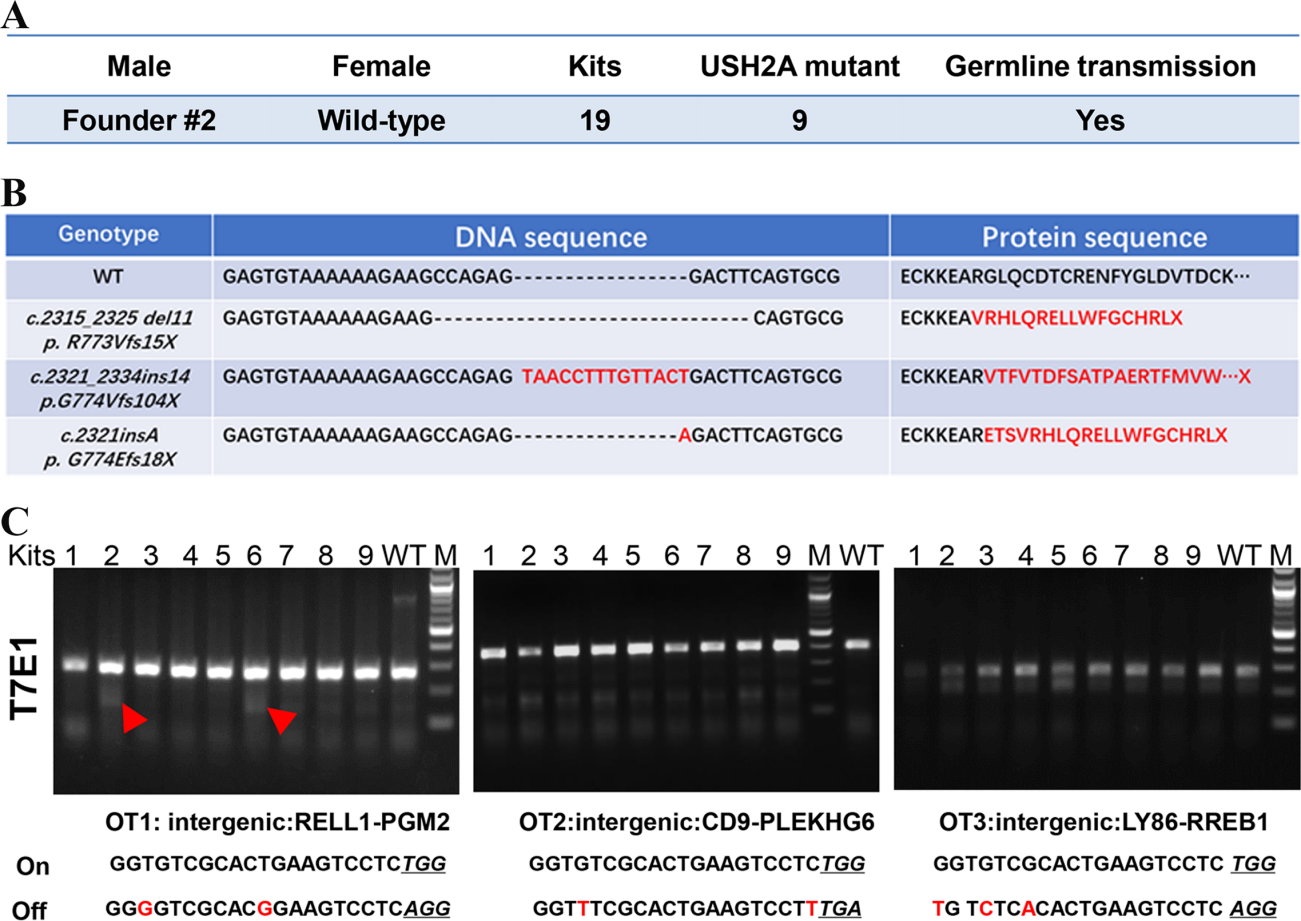
USH2A KO line establishment and off-target analysis. **(A)** Breeding of the founder male rabbit mated with two female WT rabbits. **(B)** Mutated USH2A DNA sequence and the predicted protein sequence found in the F1 generation USH2A KO rabbits. **(C)** Detection of off-target indels using T7E1 analysis in F1 generation USH2A mutant rabbits with a wild-type rabbit as control. Red arrow heads showing the indels detected at the OT1 locus in F1 generation kits #2 and #6. M, NEB 100 bp ladder DNA marker; On, on-target sequences; Off, off-target sequences; Nucleotides in red color indicate the mismatches of the gRNA and the potential off-target sequence. NGG/NGA PAMs were highlighted by underlines.

In efforts to model USH2 in rabbits, we chose to introduce frameshift mutations on rabbit USH2A exon12, equivalent to exon13 in the human genome, where the most prevalent USH2A frameshift mutation, the USH2A c.2299delG mutation is located.^5^ We designed four gRNAs targeting exon 12 of the rabbit USH2A gene (Fig. 2A). T7E1 and Sanger sequencing assay showed that sgRNA1 achieved high efficiency of cleavage in rabbit embryos (Fig. 2B). The guide RNA1 selected was co-introduced to rabbit embryos with Cas9 mRNA. In total, 60 injected embryos were transferred surgically into the oviduct of two synchronized recipients. All nine term kits were identified as USH2A mutant animals carrying high frequencies of frameshift mutations (Figs. 2C, D, Supplementary Fig. S1). These data demonstrate that USH2A mutant founder rabbits can be produced by CRISPR/Cas9 efficiently. Founder#2 was mated with two wildtype (WT) rabbits producing 19 kits, 9 of which carried frameshift insertions and deletion (indel) mutations predicted to cause premature stop codon (+14 bp, –11 bp, and –1 bp) (Figs. 3A, B).

### Off-target Analysis

To test the off-target effects, we chose the top three potential off-target sites predicted by the gRNA design software to test off-target effects, in which the predicted off-target sites were PCR amplified and analyzed with T7E1 assay and confirmed by Sanger sequencing. No indels were detected at OT2 and OT3 in any of the nine F1 rabbits (Fig. 3C), whereas at OT1, two of the nine F1 generation rabbits showed indels (GCTG to CTC) that are located 32 bp away from the predicted Cas9 cleavage site (Fig. 3C, Supplementary Fig. S2). Because this region is an intergenic region, it is more likely a natural polymorphism. Nevertheless, to avoid the potential adverse effects, these two F1 rabbits carrying the OT1 site indels were excluded from the breeding program.

### Exon 12 Mutation in Rabbits Result in Usherin KO

**Figure 4.**
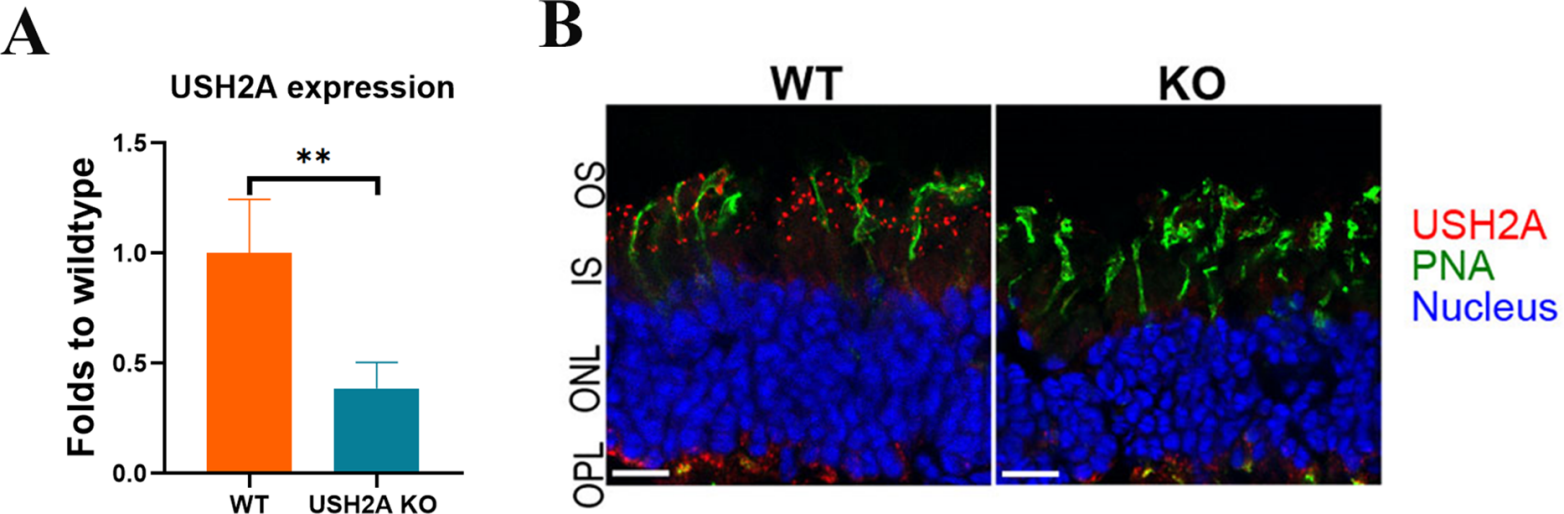
Characterization of USH2A knockout rabbits. **(A)** The relative expression level of USH2A gene in rabbit retina detected by RT-qPCR. The vertical axis shows the relative gene expression level in USH2A KO retinae (*n* = 4) relative to that in WT retinae (*n* = 3) P = 0.0065, unpaired t-test. **(B)** Immunofluorescent staining showing the expression of usherin (red) in WT retina and absent in USH2AKO rabbit retina. Cone photoreceptors were labeled with PNA (*green*) Nucleus were counterstained with DAPI (*blue*). OPL: Outer plexiform layer; IS/OS: Inner and Outer segments of rod and cone photoreceptor cells.

Upon sexual maturation, one of the F1 rabbit carrying del11 mutation (USH2A *c.2315–2325del11*) was used to establish the USH2A KO (USH2A^–/–^) line. This mutation is predicted to cause a premature stop codon, which may lead to the nonsense-mediated mRNA decay and the production of nonfunctional truncated Usherin. As shown in Figure 4A, real-time PCR detection of the USH2A transcripts in the retina shown the expression of USH2A transcripts was decreased to less than 40% that of WT controls in the USH2A KO retina, indicating that nonsense-mediated mRNA decay did happen in the USH2A KO rabbits. Immunofluorescent staining with a validated custom-made Usherin antibody indicated that Usherin are specifically expressed in outer segment of rabbit photoreceptors and are absent in the homozygous USH2A KO rabbit retina (Fig. 4B).

### Eye Phenotype of the USH2A Rabbits

**Figure 5.**
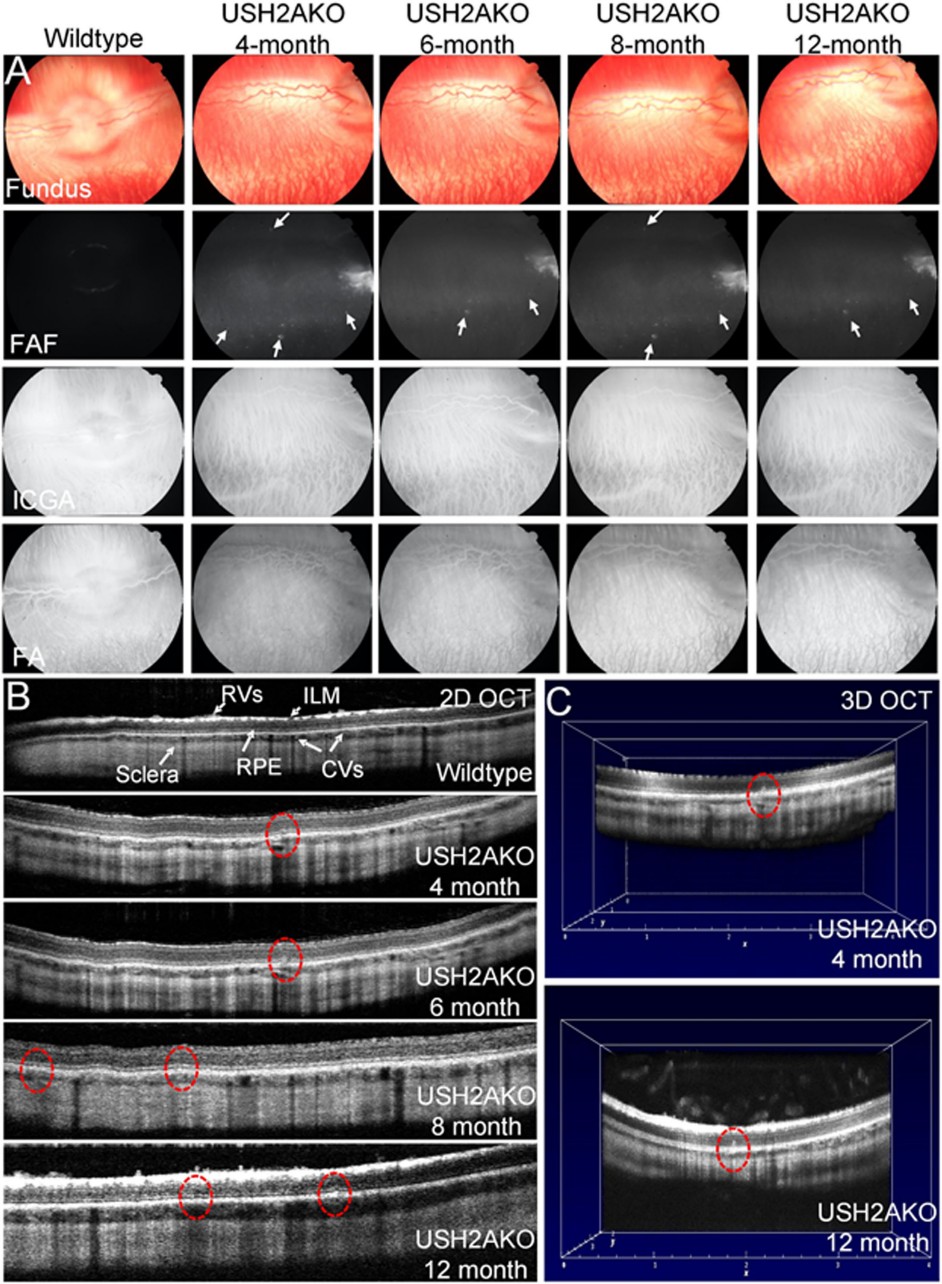
Retinal imaging in USH2A KO rabbit. **(A)** Fundus photographs (Fundus), fundus autofluorescence, indocyanine green angiography (ICGA), and fluorescein angiography (FA) images obtained from a USH2A KO rabbit at 4-month-old to 12-month-old demonstrating hyperautofluorescent spots (*arrows*). **(B)** Spectral domain OCT images of a USH2A KO rabbit at 4-month-old to 12-month-old demonstrating hyper-reflective foci at the level of the photoreceptor IS and OS segments in the photoreceptor layer (red dotted circles and red arrows in C). **(C)** Three-dimensional OCT images obtained from a USH2A KO rabbit at 4-month-old (*top*) and 12-month-old (*bottom*).

As shown in Figure 5A, USH2A KO rabbits had normal retinal and choroidal vasculature. In addition, ophthalmic examination confirmed that all USH2A rabbits had ophthalmoscopically normal and healthy corneas, anterior chambers, and clear lenses. There was no difference in the fundus appearance between WT and USH2A KO rabbits. It should be noted that these USH2A KO animals were on an albino background, and the characteristic bone spicule pigmentation of the retina seen in RP eyes would, therefore, not be expected. Fluorescein angiography and indocyanine green angiography imaging show normal retinal and choroidal vascular morphology. In contrast, hyperautofluorescent spots were detected in the retina of the USH2A KO rabbits as early as 4 months of age. OCT images also indicated changes at the photoreceptor layer with hyper-reflective foci at the level of the photoreceptor IS and OS segments as early as 4 months of age.

OCT imaging was performed on both WT and USH2A KO rabbits as illustrated in Figure 5B (B-scan) and Figure 5C (three-dimensional). B-scan OCT image obtained from WT rabbits show normal and healthy retina with the different retinal layers such as the nuclear layers, plexiform layers, photoreceptors, retinal vessels, choroidal vessels, retinal pigment epithelium (RPE), inner limiting membrane, and sclera (Fig. 5B). No evidence of photoreceptor or RPE disruption or damaged was observed in WT rabbits. In contrast, hyper-reflective foci were noted at the level of the photoreceptor inner and outer segments and RPE in USH2A rabbits at 4 months of age as marked by the red dotted circle, which increased over time with more disruptions at 8 and 12 months of age (Figs. 5B and 5C).

### Loss of Retinal Function in USH2A KO Rabbits

**Figure 6.**
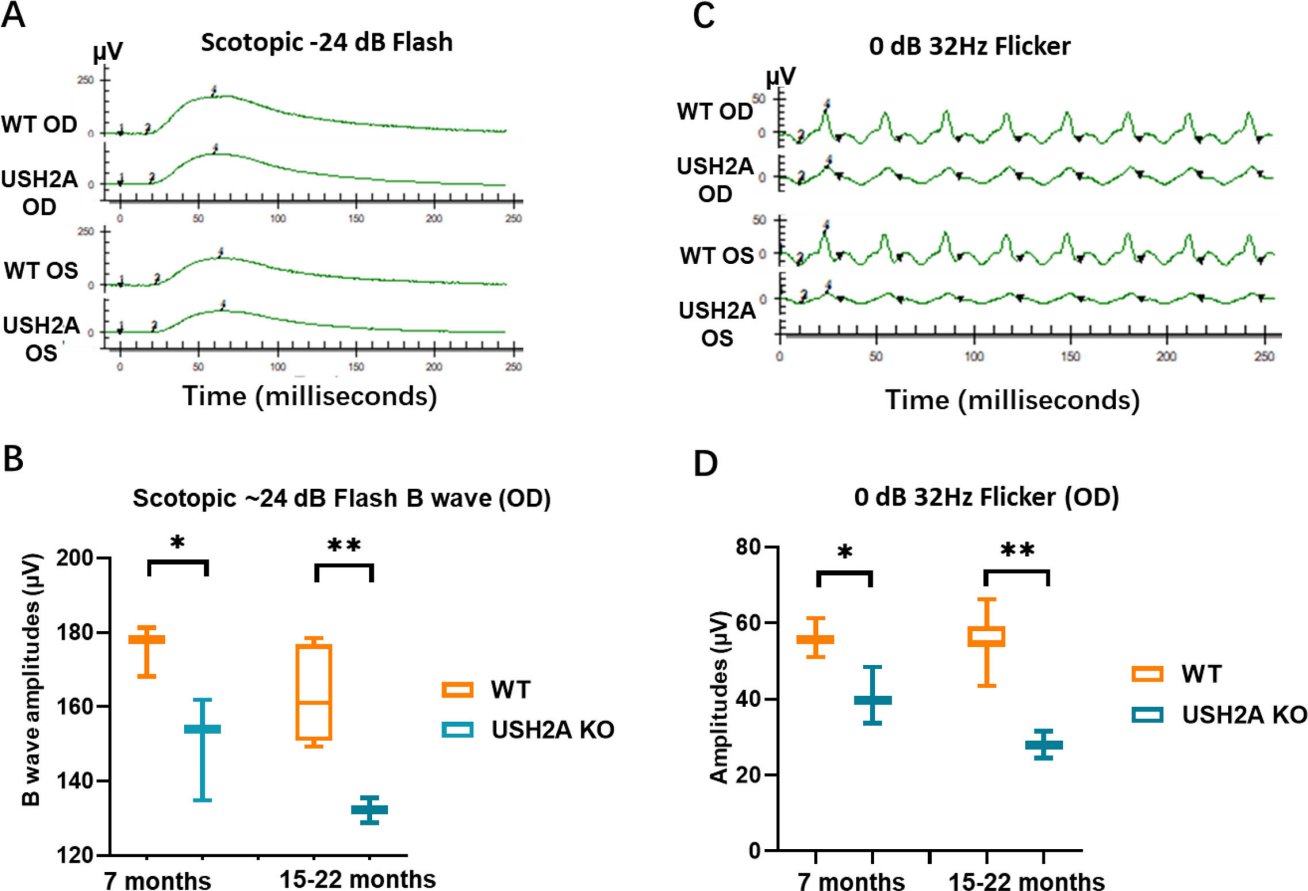
Progressive retinal degeneration in USH2A KO rabbits. **(A)** Representative rod ERGs recorded at scotopic -24 dB flash from a 15-month-old USH2A KO rabbit with an age matched wild-type rabbit as Control. **(B)** Full-field ERG demonstrated a significant reduction in rod response amplitude at 7 months that is further reduced at 15–22 months in USH2A KO rabbits compared with age matched WT rabbits (*n* = 3). **(C)** Representative ERGs recorded at 0 dB 32Hz flicker demonstrating cone response from a 15-month-old USH2A KO rabbit with an age matched wild-type rabbit as control. **(D)** A significant reduction in amplitude of cone ERGs by 7 months that is consistent and further reduced at 15–22 months were recorded in USH2A KO rabbits compared with age matched WT rabbits (*n* = 3). ICGA, indocyanine green angiography; FA, fluorescein angiography; RVs: Retinal vessels. ILM: Inner limiting membrane. CVs: Choroidal Vessels. OD: Right eye; OS: Left eye. Data analyzed by unpaired t-test, * p<0.05, ** p<0.01.

To investigate whether the disruption of USH2A gene causes retinal abnormalities, the visual function of USH2A KO and WT control rabbits were compared by ERG analyses at different ages under scotopic and photopic conditions. At 7 months of age, the USH2A KO rabbits displayed rod response (scotopic 24 dB), scotopic combined rod and cone response (scotopic 0 dB), and photopic response b-wave amplitudes (Figs. 6A, B and Supplementary Fig. 3) and 32-Hz flicker amplitudes (Figs. 6C, D) that were significantly lower than WT counterparts. There was no significant change in either a-wave amplitude or implicit time at this time point (Supplementary Figures 3–4). The responses of USH2A KO rabbits were 10% to 20% lower than those of WT control (*P* = 0.0456 and 0.042, respectively, by *t*-test) as shown in Figures 6B, 6D, and Supplementary Figure 3. At 15 to 22 months of age, scotopic b-wave amplitudes and 32-Hz flicker amplitudes became further reduced with the responses of USH2A KO more than 50% lower than those of WT controls (*P* = 0.0073 and 0.0031, respectively, by *t*-test). This age-dependent decline suggests a progressive loss of photoreceptor function in USH2A KO rabbits. Implicit time did not show a significant difference between WT and USH2A KO over time (Supplementary Fig. 4).

### USH2A Rabbit Histopathology Showed Fewer Photoreceptor Nuclei in the Outer Nuclear Layer (ONL)

**Figure 7.**
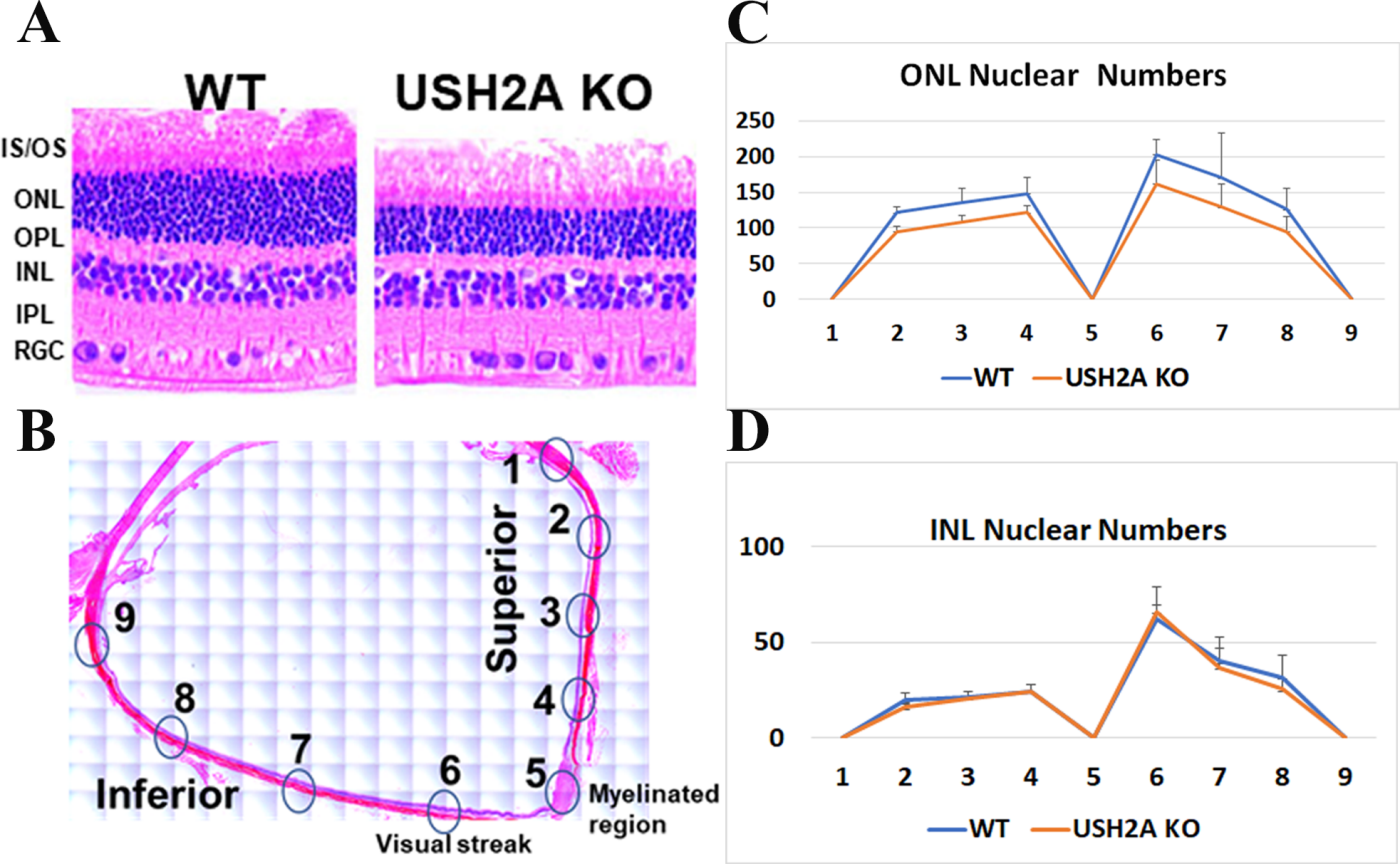
Histopathology analysis of USH2A KO rabbit retina. **(A)** Representative retinal section prepared from a 16 months USH2A KO rabbit stained with H&E show reduced retinal layer thickness compared with age-matched WT, images were token at similar locations on both retina (point 6 in B). **(B)** overview of a rabbit retinal section illustrating the locations for nuclear number counting in (C and D). **(C)** Counting of ONL layer nuclei numbers in a defined field of view (150 µm along the retina layer) at different locations on the retina indicated in panel B. **(D)** Counting of INL layer nuclei numbers in a defined field of view (150 µm along the retina layer) at different locations on the retina indicated in panel B. Error bars represent SD. RGC: Retinal Ganglion cell layer; IPL: Inner plexiform layer; INL: inner nuclear layer; OPL: Outer plexiform layer; IS/OS: Inner and Outer segments of rod and cone photoreceptor cells.

To look for photoreceptor degeneration characteristics of Usher-associated RP, the number of photoreceptor nuclei in the ONL, which represents the rod and cone cell bodies, of 16 months USH2A KO rabbits were compared with age-matched control rabbits. Figure 7A shows the H&E image of WT (left) and USH2A KO (right). These H&E images clearly show thinner ONL layer in the USH2A KO rabbit retina compared with the age-matched WT control. Figure 7B represent the overview of a rabbit retinal section illustrating the locations of optic nerve (myelinated region) and visual streak as well as the 10 spots for ONL nuclear number counting in Figure 7C. We found that the USH2A KO rabbit has fewer photoreceptor nuclear numbers throughout the whole retina compared with the age-matched controls, indicating that photoreceptor cell degeneration did happen at least by 16 months of age, which explained the reduced ERG signals in Figure 6. The counting of inner nuclear layer nuclear numbers did not find a significant difference between WT and USH2A KO rabbits (Fig. 7D).

### USH2A KO Rabbits Showed Moderate to Severe Hearing Loss

**Figure 8.**
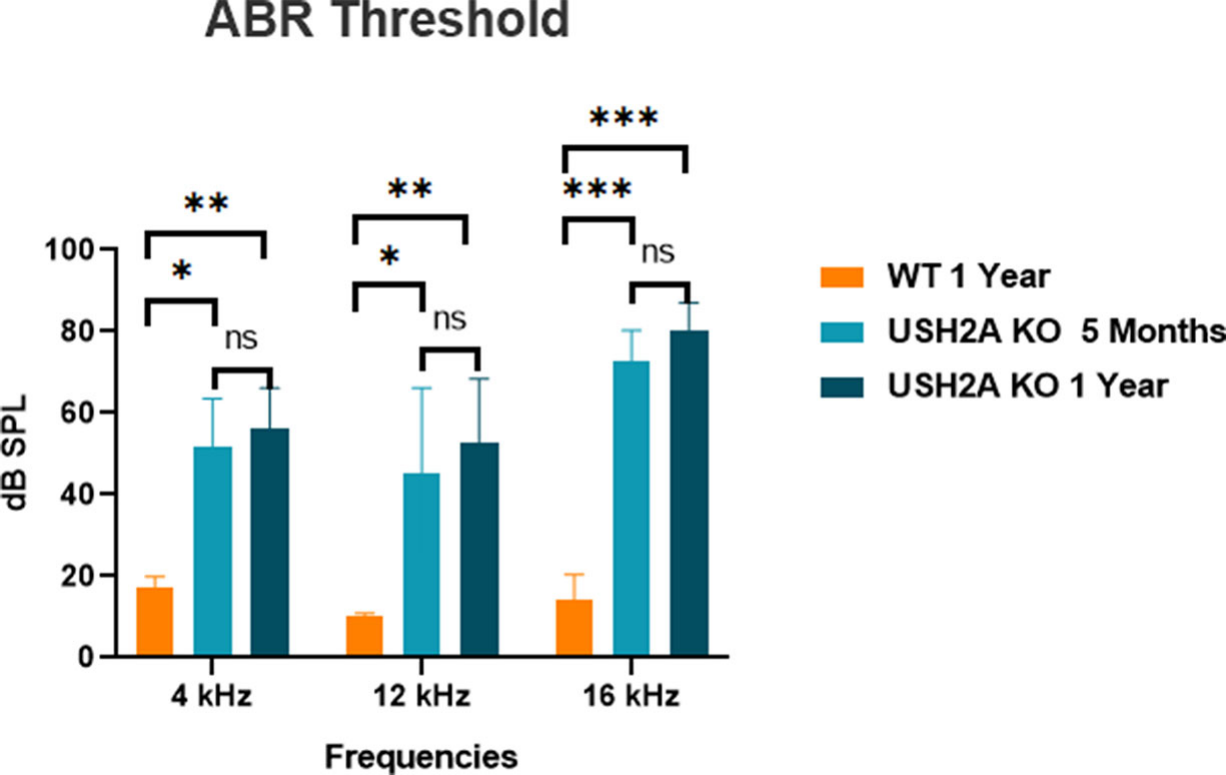
Hearing loss in USH2A KO rabbits. ABR test demonstrating significant hearing loss in the USH2A KO rabbits at 5 and 12 months old compared with WT control rabbits at 12 months old in all three frequencies (4 kHz, 12 kHz and 16 kHz) tested. No difference was found between the USH2A KO rabbits at 5 months and 12 months old group (3 animals per group, data analyzed by 2-way ANOVA).

USH2 affects both hearing and vision. In this study, ABR was tested in three USH2A KO rabbits at 5 months and 1 year of age. As shown in Figure 8, the ABR threshold at three different frequencies tested (4, 12, and 16 kHz) were all increased in the USH2A KO rabbits (43–85 db sound pressure level) compared with WT control rabbits at 12 months old (10–20 db sound pressure level), indicating moderate to severe hearing loss in these rabbits that develops slowly over time.

USH type 2 is characterized by hearing loss and early adulthood-onset of RP. Retinal degeneration in patients is apparent by fundus examination and the progressive reduction in ERG amplitudes over the course of the disorder. A targeted Ush2a KO mouse demonstrates only mild retinal degeneration with late age of onset.^6^ A spontaneous mutant mouse model, Kunming, shows a rapid, early-onset retinal degeneration, but contains mutations in two genes known to be involved in inherited retinal dystrophies, namely, Ush2a and Pde6b.^41^ Recently, bright light induction has been reported to be able to induce the damage of the rod photoreceptors in several of the USH1 and USH2 mouse models. However, these experiments were in a 129 Sv/j background, which is inherently more sensitive to light-induced photoreceptor cell damage.^42^^,^^43^ Overall, these models only showed slightly reduced rod function indicated by reduced scotopic b wave amplitude, but no cone function reduction was detected. This is probably due to their late onset features; cones are affected later in the disease course compared with rods. Owing to the short life span of the mice, it is very difficult to study USH2 disease in mouse models. Here, we have successfully established an USH2A KO rabbit line. We expect the frameshift mutation will produce a loss-of-function truncated Usherin protein that is similar to that from the predominant USH2A c.2299delG mutation, which is also a frameshift mutation located 16 bp upstream. To date, all the disease-causing mutations found in USH2A are recessive and loss-of-function mutations. No dominant negative mutation has been found among more than 700 USH2A mutations reported. In addition, we did not see any visual or hearing defects in the heterozygous rabbits (data not shown), indicating this mutation is not dominant negative. We found that targeted disruption of the USH2A gene in rabbits resulted in hearing loss and retinal degeneration starting as early as 4 to 7 months of age, which is equivalent to human adolescence, and showed progressive retinal degeneration mimicking the RP in USH2 patients. To our knowledge, it is the first genetic preclinical large animal model that manifest eye phenotype of USH2.

It should be noted that these USH2A KO animals were on an albino background, which may affect the manifestation of the RP phenotype. Classic retina degeneration phenotype have been successfully studied in rhodopsin Pro347Leu transgenic rabbits, a model of RP in both NZW and Dutch Belted background, with the model in NZW background have been used more frequently.^20^^–^^31^ In this study, we found that USH2A mutant NZW rabbit shown the expected visual loss and photoreceptor degenerations mimicked the RP symptoms found in human patients. It is known that oculocutaneous albinism is caused by mutations in single gene encoding tyrosinase (TYR). CRISPR/Cas9–mediated repair of the albinism-associated TYR K373T SNP in rabbits exhibited rescued melanin production in rabbits.^44^ The TYR gene in rabbit is located on chromosome1, whereas the USH2A gene in rabbits is located on chromosome 16. We may consider breeding this model with pigmented rabbits to establish an USH2A mutant pigmented rabbit line to study the characteristic bone spicule pigmentation of the retina seen in RP eyes.

In humans, the location of highest acuity in the retina is a circular area termed the fovea, which resides the highest concentration of cones, the lowest concentration of rods, and much smaller receptive field sizes for all cells. The area of greatest acuity in rabbit retina is not a single point, but rather an elongated “streak” running across the retina. The presence of the visual streak made rabbits very useful for studying retinal degenerative diseases in contrast to mice. As shown in Figure 7, the decrease in the ONL nuclear numbers in USH2A KO rabbits were more apparent in the visual streak area (point 6), suggesting a loss of cone photoreceptors. It is reported that severe visual phenotype seen in syndromic USH2A patients compared with the nonsyndromic USH2A patients could relate to a greater extent of cone dysfunction indicated by significantly reduced 30-Hz flicker ERG amplitudes.^45^ In this study, in addition to the decreased rod function detected by the decreased scotopic b-wave amplitudes in ERG, our USH2A KO rabbit models also showed significantly decreased 32-Hz flicker ERG amplitudes that mimic the cone function reduction in human patients.

In human patients, USH2 is characterized by congenital moderate to severe hearing loss. We observed moderate hearing loss in the USH2A rabbit models and there is no significant change between 5 and 12 months of age in hearing function. We planning to do a natural history study on this model to further confirm that the hearing loss is congenital.

## Conclusions

We have succeeded in generating a rabbit model of USH2. Although further studies are needed to fully characterize the natural history of hearing loss and retinal degeneration phenotype in USH2A KO rabbits and to determine the exact mechanism of photoreceptor dysfunction observed in this model, we believe that this USH2A mutant rabbit model will serve as a useful large animal model with which to study the pathophysiology of RP in USH and develop novel treatments. The successful replication of the RP phenotype in USH2A rabbit models in this study as well as the extension of gene targeting technology to rabbits by CRISPR/Cas9 technology motivating efforts to develop rabbit models for other types of USH, as well as other hereditary retinal diseases.

## Supplementary Material

Supplement 1
