# Supplementary material for: USH2A Gene Mutations in Rabbits Lead to Progressive Retinal Degeneration and Hearing Loss
Source: Transl Vis Sci Technol. 2023 Feb 16;12(2):26. doi: 10.1167/tvst.12.2.26 (PMC9940772; doi:10.1167/tvst.12.2.26)
Supplement: Supplement 1 [file tvst-12-2-26_s001.docx]

**Supplementary Information******

**USH2A gene mutations in rabbits lead to progressive retinal degeneration and hearing loss**

**Van Phuc Nguyen^2#^, Jun Song^1#^, Diane Prieskorn^3^, Yanxiu Li^2^, David Dolan^3^, Jie Xu^1^, Jifeng Zhang^1^, K Thiran Jayasundera^2^, Yehoash Raphael^3^, Y. Eugene Chen^1^, Yannis M. Paulus^2*^, Dongshan Yang^1*^**

^1^Center for Advanced Models for Translational Sciences and Therapeutics, University of Michigan, Ann Arbor, MI 48109, USA

^2^Kellogg Eye Center, Department of Ophthalmology and Visual Sciences, University of Michigan, Ann Arbor, MI 48105, USA

^3^Kresge Hearing Research Institute, Department of Otolaryngology-Head and Neck Surgery, University of Michigan, Ann Arbor, MI 48109, USA

# These authors contributed equally to this work.

^*^Corresponding Author:

Yannis M. Paulus, M.D., F.A.C.S.

Department of Ophthalmology and Visual Sciences

Department of Biomedical Engineering

University of Michigan

1000 Wall Street

Ann Arbor, MI 48105, USA

Email Address: ypaulus@med.umich.edu

Dongshan Yang, PH.D.

Center for Advanced Models for Translational Sciences and Therapeutics Department of Internal Medicine

University of Michigan

2800 Plymouth Rd NCRC B26-355S

Ann Arbor, MI 48109-2800, USA

1000 Wall Street

Ann Arbor, MI 48105, USA

Email Address: doyang@med.umich.edu


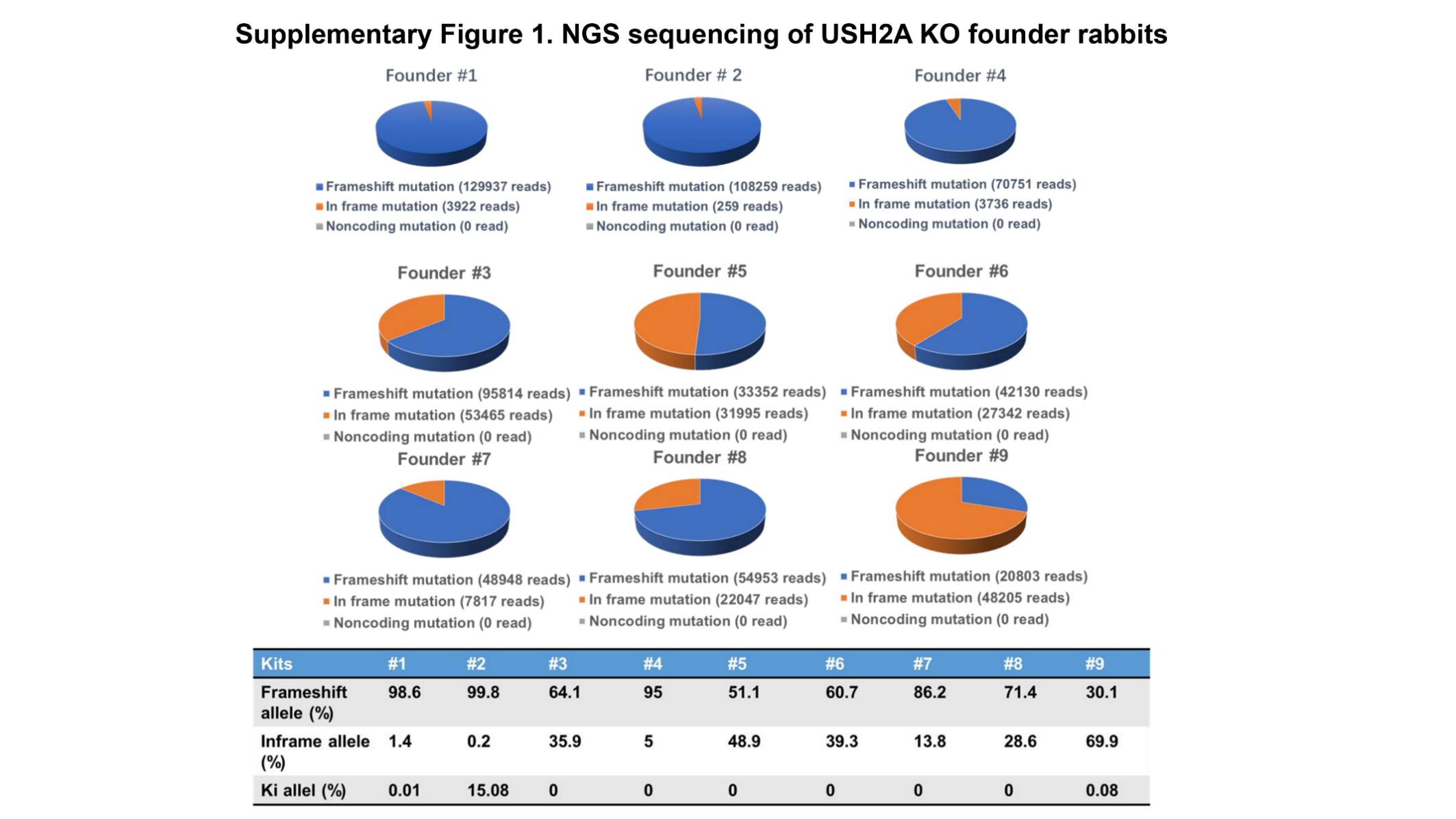


**Figure S1: Mutations found in USH2A KO founder rabbits.** The sgRNA target sequence were PCR amplified and sequenced by Next generation sequencing (NGS) and analysed by online software CRISPResso2. Frequencies of mutations in each founder animal were indicated.


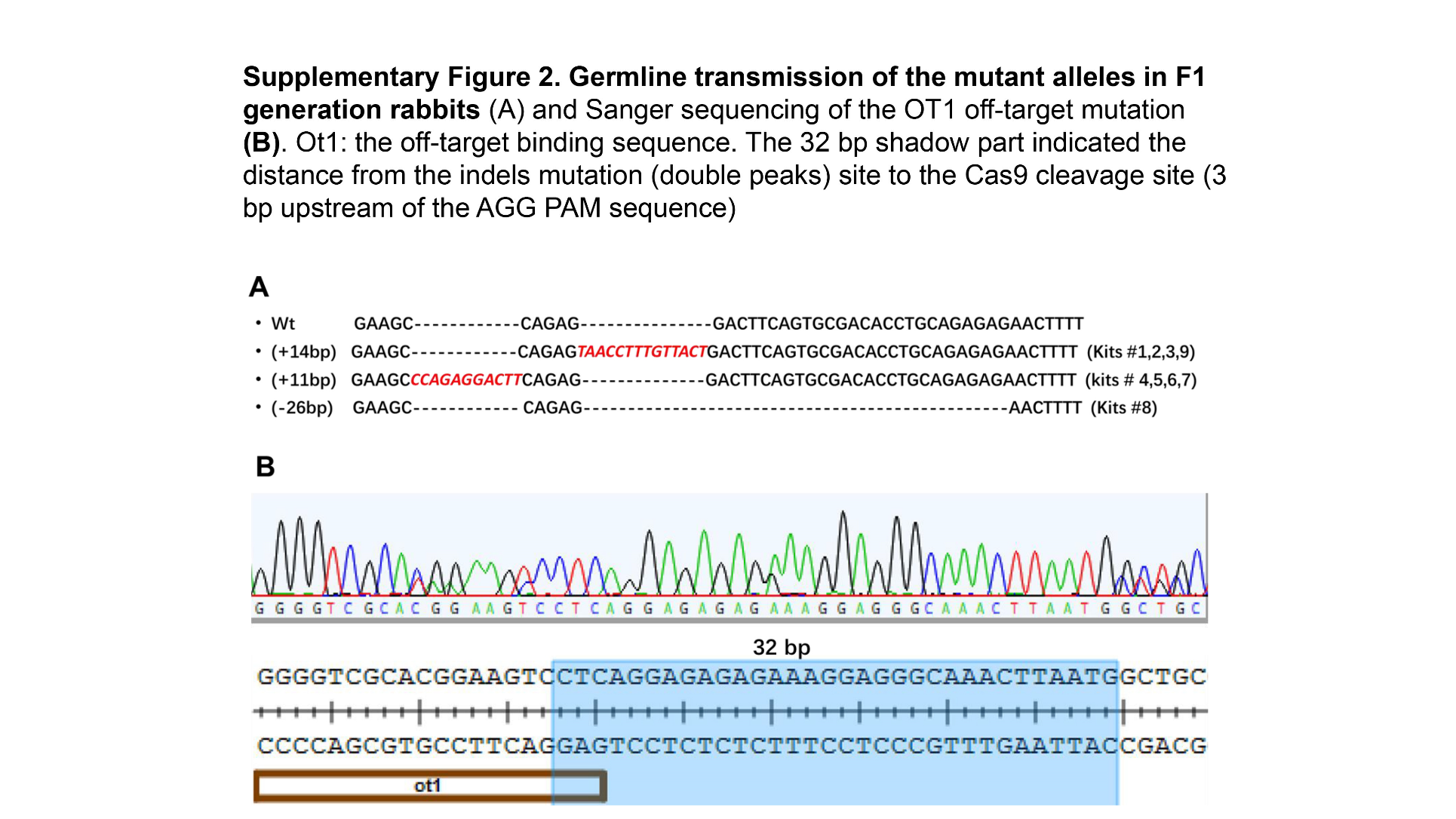


**Figure S2: Genotyping of F1 generation rabbits.** (A). Germline transmission of the mutant alleles in F1 generation rabbits detected by Sanger sequencing. (B). Sanger sequencing of the OT1 off-target mutation. Ot1: The off-target binding sequence. The 32 bp shadow part indicate the distance from the indels mutation (double peaks) site to the Cas9 cleavage site (3 bp upstream of the AGG PAM sequence)


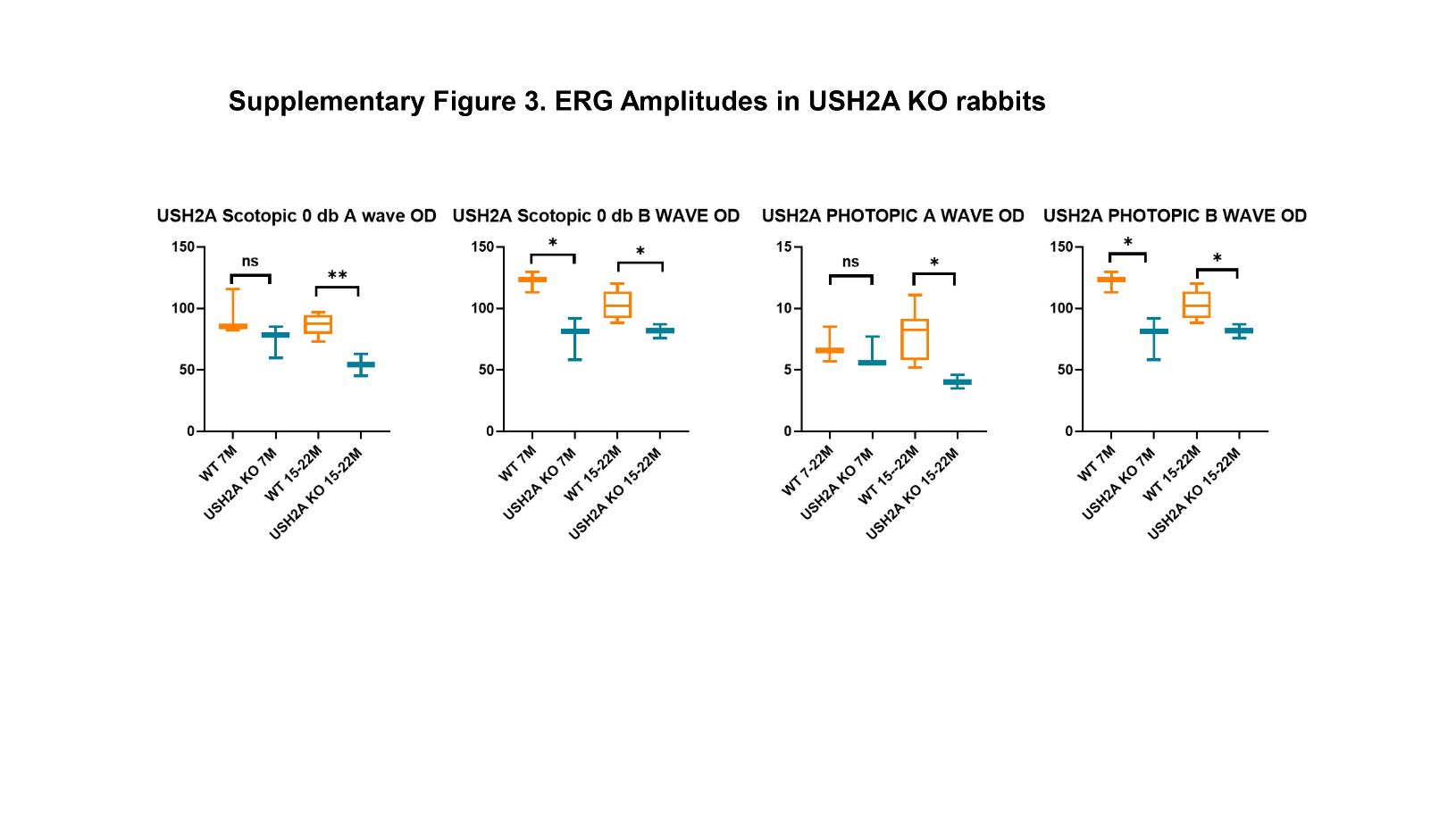


**Figure S3: ERG amplitudes in USH2A KO rabbits.** A and B wave amplitudes of field electroretinography (ERG) recorded under different adaptation conditions in USH2A KO rabbits with age matched wildtype (WT) rabbits as control. Only the data from right eyes (OD) were compared and analysed by unpaired t-test, * p<0.05, ** p<0.01.


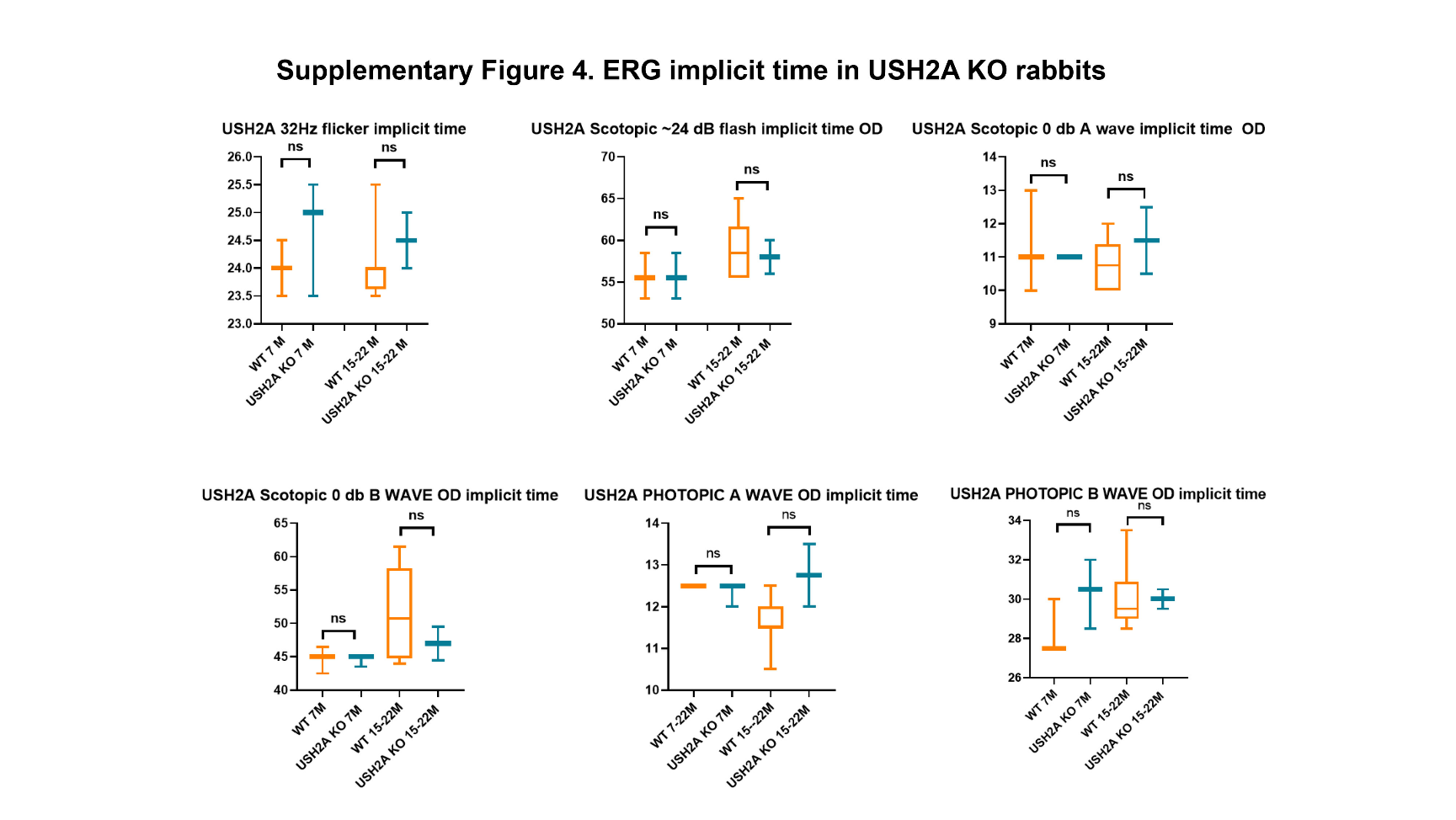


**Figure S4: ERG implicit time in USH2A KO rabbits.** Implicit time of field electroretinography (ERG) were recorded under different adaptation conditions in USH2A KO rabbits with age matched wildtype (WT) rabbits as control. Only the data from right eyes (OD) were compared and analysed by unpaired t-test, * p<0.05, ** p<0.01.


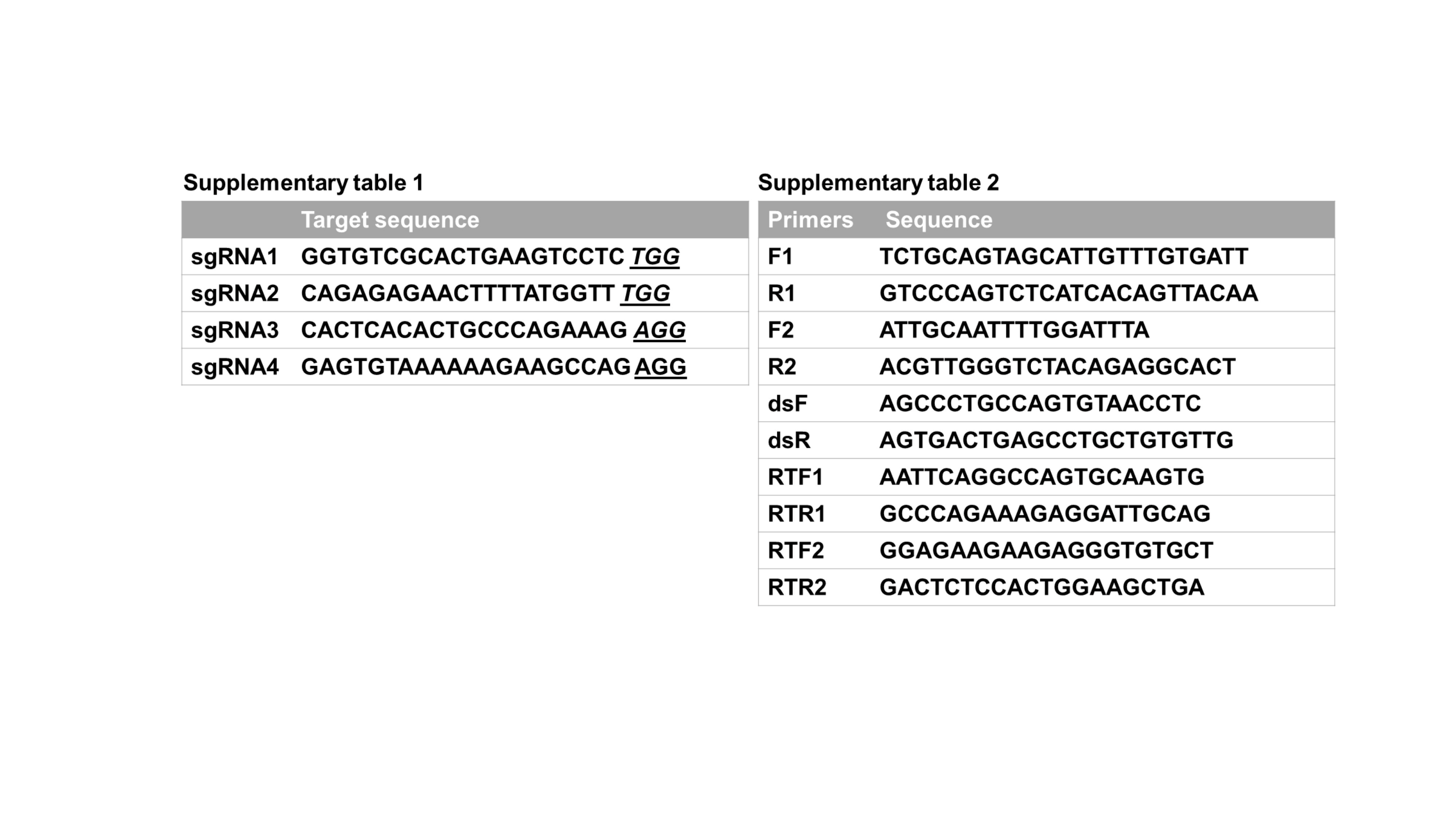


**Table S1: Design of single guide RNA targeting rabbit USH2A exon 12**. The NGG PAM sequences were highlighted by italic underlined.

**
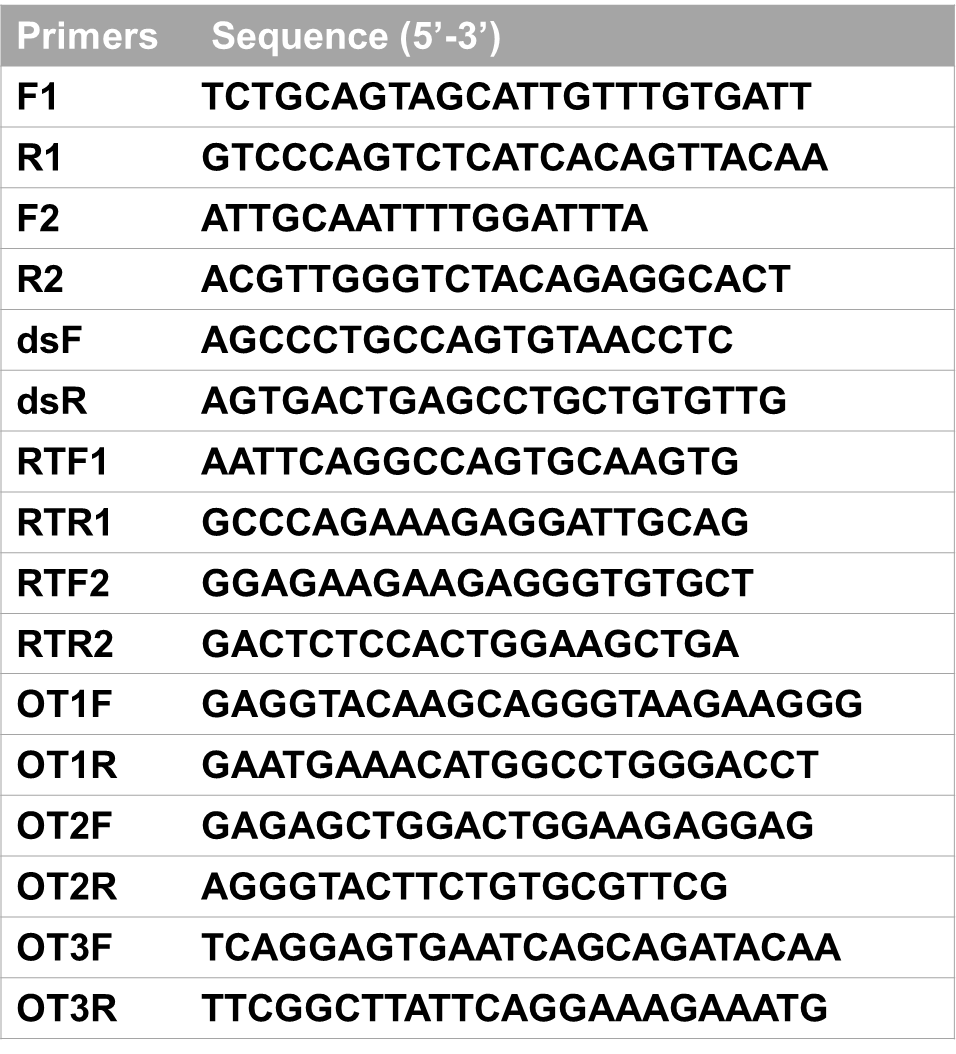
**

**Table S2: Primers used for genotyping and Realtime PCR in this study.**
